# Proteomic analysis of sperm from fertile stallions and subfertile stallions due to impaired acrosomal exocytosis

**DOI:** 10.1038/s41598-024-63410-3

**Published:** 2024-05-30

**Authors:** Camilo Hernández-Avilés, Luisa Ramírez-Agámez, Susan T. Weintraub, Charles F. Scoggin, Brian W. Davis, Terje Raudsepp, Dickson D. Varner, Charles C. Love

**Affiliations:** 1 Equine Fertility Laboratory, Department of Large Animal Clinical Sciences, School of Veterinary Medicine and Biomedical Sciences, Texas A&M University, 500 Raymond Stotzer Parkway, College Station, TX 77843 USA; 2 Veterinary Integrative Biosciences, School of Veterinary Medicine and Biomedical Sciences, Texas A&M University, College Station, TX USA; 3 Department of Biochemistry and Structural Biology, The University of Texas Health Science Center, San Antonio, TX USA; 4 LeBlanc Reproduction Center, Rood & Riddle Equine Hospital, Lexington, KY USA

**Keywords:** Stallion sperm, Thoroughbred, Impaired acrosomal exocytosis, Proteomics, Acrosome enzymes, Arylsulfatase F, Proteomics, Animal physiology, Translational research

## Abstract

Thoroughbred stallions that carry a double-homozygous genotype A/A-A/A for SNPs rs397316122 and rs69101140 in exon 5 of the *FKBP6* gene (chr13; EquCab3.0) are uniquely subfertile due to impaired acrosomal exocytosis (IAE). In this study, the sperm proteome in frozen/thawed semen from subfertile Thoroughbred stallions was studied and compared to that of frozen/thawed sperm from fertile Thoroughbred stallions. A total of 2,220 proteins was identified, of which 140 proteins were found to be differentially abundant in sperm from the subfertile stallions compared to that of fertile stallions (83 less and 57 more abundant). Proteins of differential abundance in sperm from the subfertile stallions were mainly overrepresented in the “metabolism” and the “metabolism of lipids” pathways. One of these proteins, arylsulfatase F (ARSF), was studied by immunofluorescence. A lower proportion of sperm displaying ARSF signal at the acrosome region was observed in sperm from subfertile Thoroughbred stallions. In addition, heterologous zona pellucida binding assays revealed that sperm from subfertile Thoroughbred stallions bound at a lower proportion to zonae pellucidae than sperm from fertile Thoroughbred stallions. In conclusion, a group of differential abundance proteins, including some of acrosome origin, were identified in sperm from subfertile stallions with acrosome dysfunction.

## Introduction

The acrosomal exocytosis (AE) process involves a series of biochemical changes in the sperm, mediated mainly through increased intracellular pH and calcium levels and plasma membrane destabilization due to cholesterol depletion^1–3^. Together, all these cellular changes will result in the fusion of the outer acrosomal membrane with the sperm plasma membrane, leading to the release of multiple enzymes at the vicinity of the cumulus-oocyte complex (COC) and facilitating the binding of sperm to the zona pellucida^1–3^. Stallion subfertility due to impaired acrosomal exocytosis (IAE) is a condition identified thus far only in stallions of the Thoroughbred (TB) registry. Sperm from these stallions are characterized as having a lower acrosomal response after in vitro exposure to either non-physiologic (i.e., calcium ionophore A23187) or more physiologic (i.e., lactate-induced) conditions known to result in acrosomal exocytosis (AE)^4–6^. These stallions have low fertility (< 30% per-cycle pregnancy rates), even though typical features of sperm quality and breeding management are acceptable. An association between the IAE phenotype and the presence of a double homozygous A/A-A/A genotype for SNPs chr13:11,353,372G > A (rs397316122) and chr13:11,353,436A > C (rs69101140) in *FKBP6* exon 5 (EquCab3) has been identified in two separate studies^7,8^. The frequency of subfertile TB stallions that carry the *FKBP6* A/A-A/A genotype approaches 1–3% of the TB breeding stallion population (four out of 150 stallions evaluated in Central Kentucky, USA, for the presence of the susceptibility genotype^8^; and seven out of 1128 stallions of various breeds evaluated during 17 years at a reference laboratory^5^). In mice, the FKBP6 protein has been associated with the normal formation of the synaptonemal complex during spermatogenesis; thus, double knock-out male mice are sterile due to azoospermia^9^. Similar findings have also been identified in men with idiopathic azoospermia^10,11^, indicating that FKBP6 is related to the normal progression of meiosis in spermatocytes. These findings contrast the clinical characteristics of TB stallions with the A/A-A/A combined genotype in *FKBP6* exon5 (i.e., normal sperm quality and testicular size), making a potential link between the susceptibility genotype for IAE in these stallions and the function of FKBP6 challenging to demonstrate.

Proteomic technologies have been used to identify proteins of importance for sperm physiologic processes^12–16^ and to identify candidate biological markers that could either be used to select males with higher fertility potential^17–19^ or identify potential proteins that explain causes of reduced fertility^20–22^. Most of these studies included proteomic analysis using mass spectrometry-based technologies, mainly involving liquid chromatography coupled with tandem mass spectrometry (LC–MS/MS). In this method, the mass-to-charge ratio (*m/z*) of ionized molecules (in this case, peptides produced by proteolytic digestion during sample preparation) are detected, and the ions then fragmented, generating precursor (MS1) and product-ion (MS2) mass spectra^23,24^. These MS2 mass spectra are then queried against published protein sequence databases to identify (and relatively quantify) the proteins in the sample^23,24^. The mass spectra produced in this way are obtained using a data-dependent acquisition (DDA) approach, in which peptides detected in a precursor scan are sequentially selected based on relative abundance and fragmented to generate sequence-informative tandem mass spectra^25–27^. When using DDA-MS to analyze complex samples, there is inevitably under-sampling (i.e., lack of detection and fragmentation of many lower-abundance peptides) even with state-of-the-art instruments with extremely fast scan rates. This can hamper the detection of low-abundance proteins which may have biological relevance^24,28,29^. Recently, a data-independent acquisition mass spectrometry (DIA-MS) approach has gained popularity. In DIA-MS, ionized peptides in small “windows” of m/z ranges are sequentially fragmented rather than individually selected precursors, thereby permitting much more comprehensive detection of the complement of peptides in a digest^30–33^. In this way, DIA-MS provides identification and relative quantification of a much larger number of proteins with greater accuracy and precision than DDA-MS^30,33^.

Various studies have analyzed the proteome of sperm following incubation under capacitating conditions or after attempted stimulation of AE^13,34,35^. To our knowledge, such studies have not yet been conducted using stallion sperm. We recently reported a method to consistently induce AE in sperm from stallions with different in vivo fertility levels, including TB stallions confirmed to have IAE by genotyping and acrosomal function testing^6^. Given the potential advantages of DIA-MS to identify candidate proteins that otherwise would not be discovered by other methods, we combined this approach with our method to induce AE in stallion sperm to identify potential candidate proteins that could explain the etiology of IAE in TB stallions.

## Results

### Sperm quality parameters in fresh and frozen/thawed semen from fertile and subfertile TB stallions (A/A-A/A)

**Figure 1:**
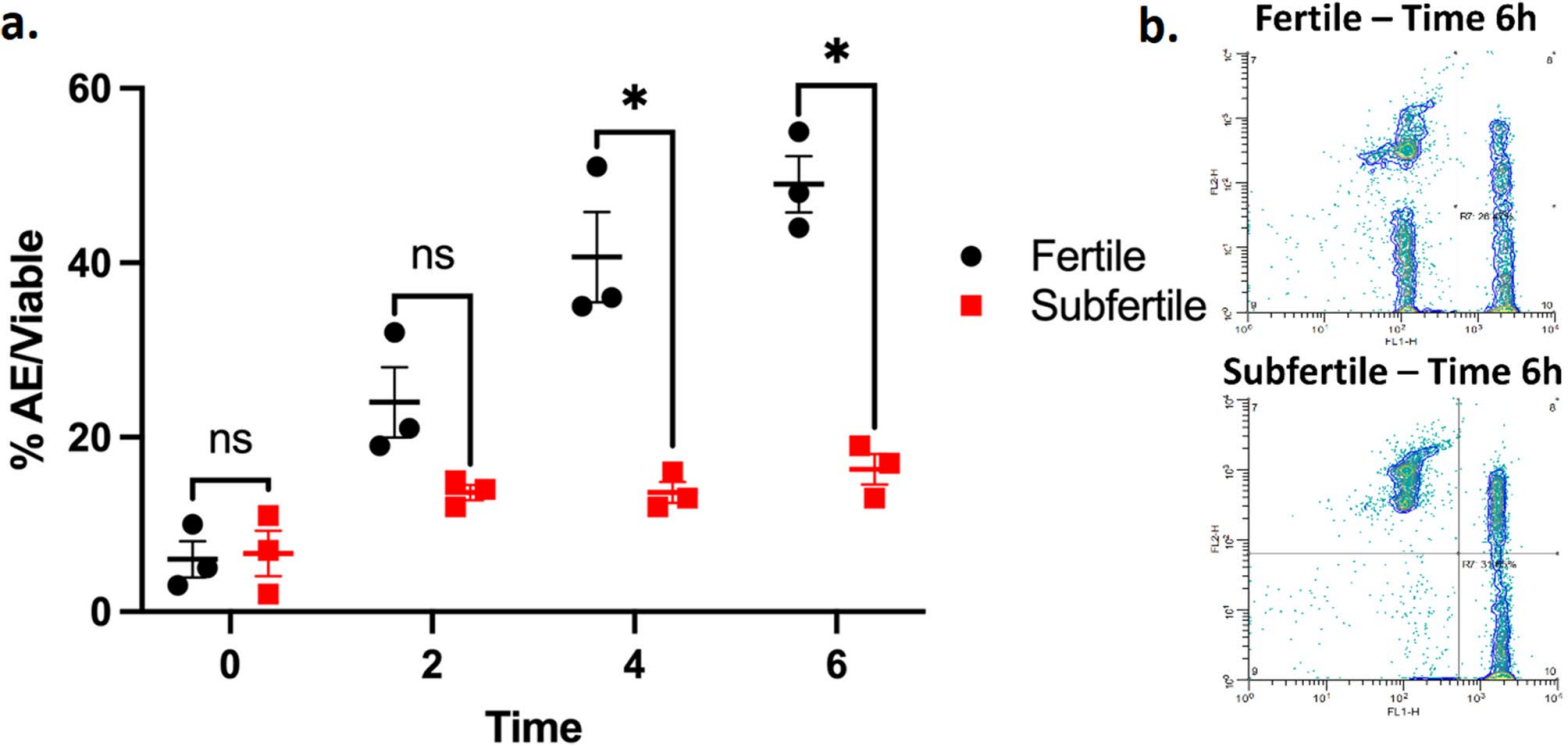
**a** The mean (± SEM) percent of AE in viable sperm of frozen/thawed semen from three fertile (n = 3) Thoroughbred stallions (black dots) and three subfertile (n = 3) Thoroughbred stallions (red squares) that carry the susceptibility genotype for IAE. Frozen/thawed semen was processed using the Lac-MW model for induction of AE. Data are presented as mean (black bar) ± SEM. **b** Representative scatterplots of the flow cytometric evaluation of viability/acrosomal exocytosis at 6 h incubation in Lac-MW are presented. The fluorescent signal of FITC-PSA is represented on the X-axis of the scattergrams, while on the Y-axis, the fluorescent signal of the Fixable Live/Dead Red Stain is represented. Within each period (T0h, T2h, T4h, T6h), the asterisk (*) indicates significant differences between stallion groups (fertile vs. subfertile) (*P* < 0.05).

The initial sperm quality parameters in fresh semen, fertility parameters (mare book, PC-PR, and SPR), and the *FKBP6* genotype results from both stallion groups are shown in Supplementary Table 1. No differences were observed in sperm quality between stallion groups (*P* > 0.05). Frozen/thawed semen from both fertile and subfertile TB stallions was exposed to the Lac-MW model to determine their acrosomal response. At T0h and T2h, the mean (± SEM) AE/Viable was similar for fertile and subfertile stallions (4 ± 2% vs. 5 ± 2% and 23 ± 5% vs. 16 ± 2%, respectively. Figure 1; *P* > 0.05), while at T4h and T6h, the mean AE/Viable was higher for fertile than for subfertile stallions (41 ± 6% vs. 17 ± 2% and 49 ± 4% vs. 19 ± 3%, respectively; *P* < 0.05). Representative scattergrams of the viability/acrosomal exocytosis assay conducted in frozen semen from a fertile stallion and a subfertile A/A-A/A stallion are also presented in Fig. 1.

### The proteome of frozen/thawed sperm from fertile and subfertile TB stallions

**Figure 2:**
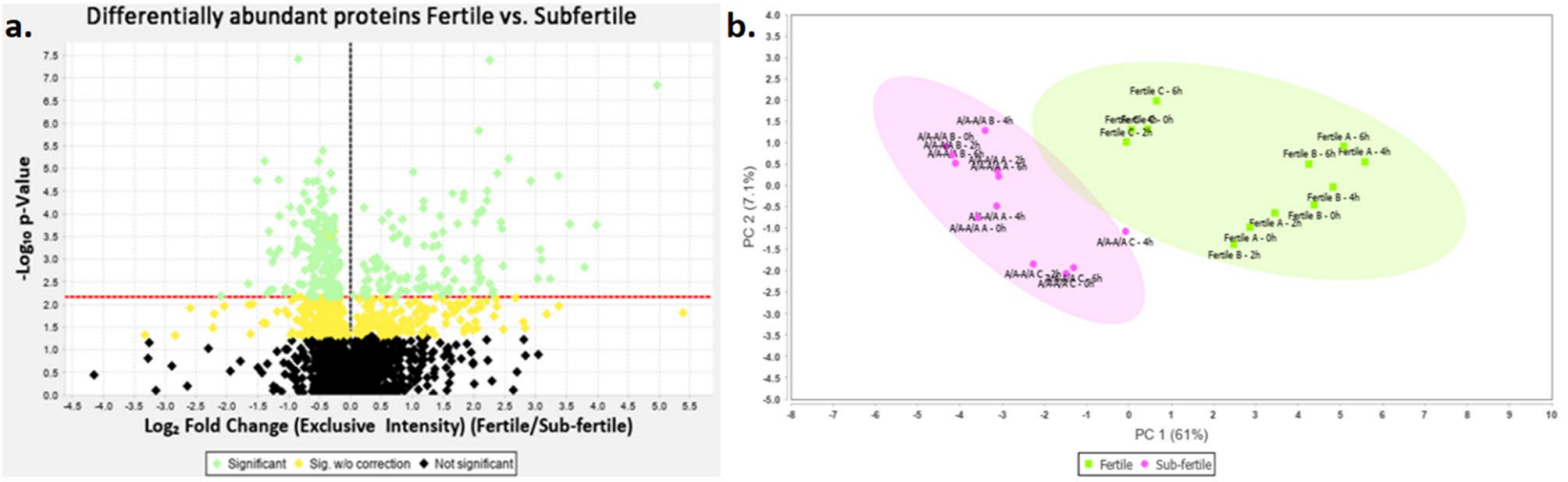
**a** Volcano plot illustrating the differences in relative protein abundance in sperm from fertile and subfertile Thoroughbred stallions. The *x*-axis denotes the log_2_(fold change) between stallion groups (fertile vs. subfertile), while the *y*-axis represents the − log_10_ *P*-value of the change in protein abundance between stallion groups (fertile vs. subfertile). **b** A principal component analysis (PCA) of the proteins of differential abundance (140 proteins; two-way ANOVA, q-value 0.05; log_2_fold <  − 0.586 > 0.586) between stallion groups (fertile vs. subfertile) and among periods (T0h, T2h, T4h, T6h), is presented, whereby a clear clustering of the stallion groups can be appreciated. Green diamonds—Significant: two-way ANOVA with Benjamini–Hochberg multiple testing correction (FDR q-value 0.05). Yellow diamonds—Significant (*P* < 0.05) without multiple testing correction. Black diamonds—Not significant.

Two thousand two hundred two (2,202) proteins (FDR 1.0%) were identified from 18,723 peptides by DIA-MS in sperm from both fertile and subfertile TB stallions. Using the stallion phenotype (fertile vs. subfertile) as the main effect to test by two-way ANOVA, 298 proteins reached significance after FWER correction (FDR q-value 0.05; Fig. 2). After applying stringent criteria (i.e., log2fold ≤  − 0.585 or ≥ 0.585), a total of 140 proteins were identified, which identified 61% of the variance in protein abundance between stallion groups, resulting in two distinctive data point clusters (Fig. 2). Of these, 83 were found to be of lower relative abundance in sperm from the subfertile TB stallions [log_2_(fold change) <  − 0.585]. In comparison, 57 proteins were found to have a higher relative abundance [log_2_(fold change) > 0.585] in sperm from these stallions when compared to sperm from fertile TB stallions. The 140 differentially abundant proteins were used for subsequent analysis.

### Gene ontology analysis of lower or higher abundance sperm proteins in subfertile TB stallions

**Figure 3:**
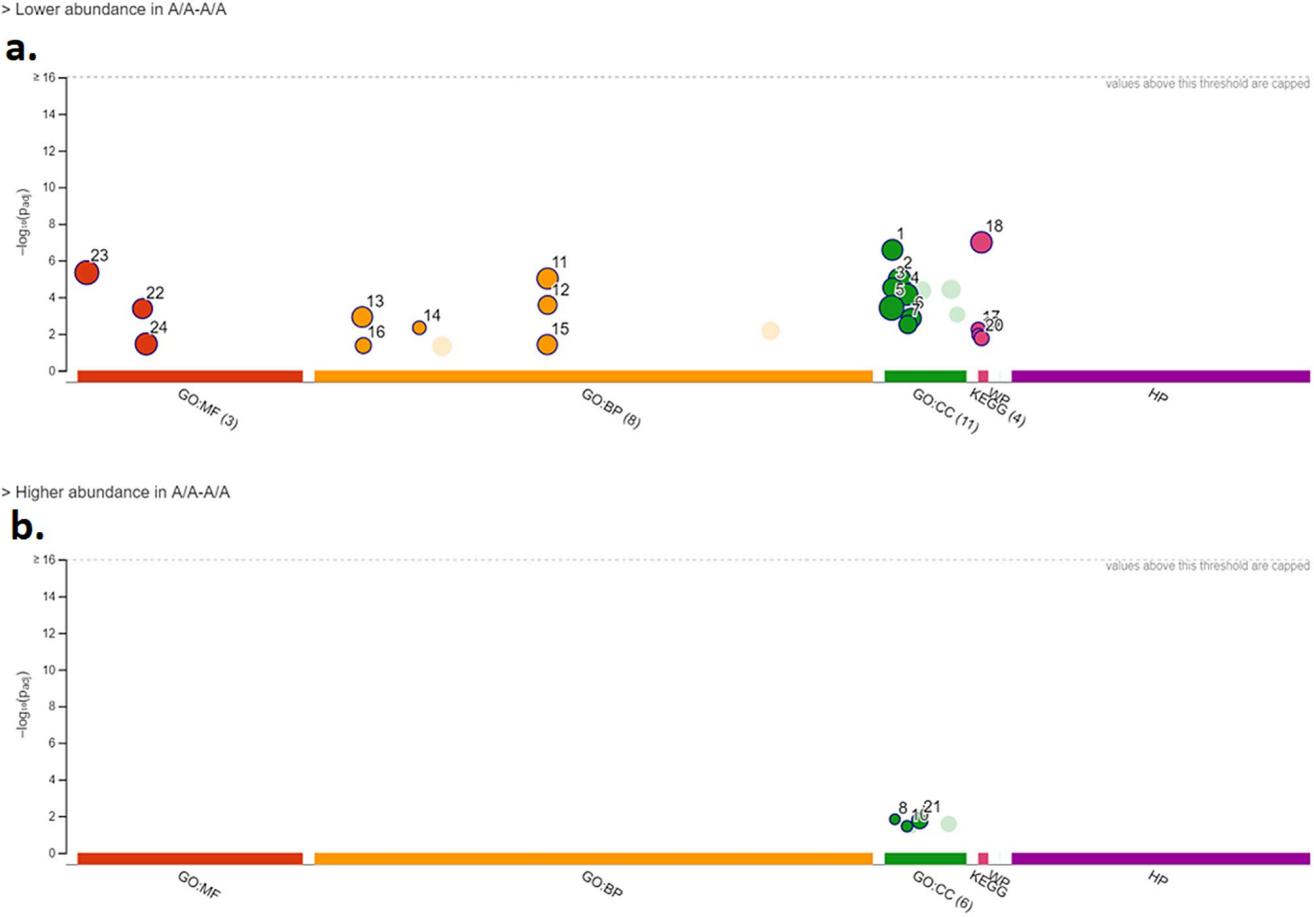
g: Profiler (g: GOST) multi-query Manhattan plot demonstrating the enrichment analysis of differentially abundant proteins (lower [**a**] and higher [**b**] relative abundance) in sperm from subfertile TB stallions when compared to sperm from fertile TB stallions. The proteins were queried using *Equus caballus* orthologs. The adjusted *p*-values for statistical overrepresentation are depicted on the y-axis of each plot. *GO* Gene ontology; *CC* cellular component; *BP* biological process; *MF* molecular function; *KEGG* Kyoto encyclopedia of genes and genomes.

An initial analysis using g:Profiler and *Equus caballus* orthologs was conducted in each one of the two lists of proteins of lower and higher abundance in sperm from subfertile TB stallions when compared to fertile TB stallions. Manhattan plots corresponding to the Gene Ontology (GO) terms Cellular Component (CC), Biological Process (BP), Molecular Function (MF), and Kyoto Encyclopedia of Genes and Genomes (KEGG) analyses are presented in Fig. 3. On the other hand, GO terms for each of these categories in proteins of lower and higher abundance in sperm from subfertile stallions are presented in Table 1. Subsequent GO analyses were conducted in g:Profiler by using *Homo sapiens* rather than *Equus caballus* orthologs to increase the coverage of GO terms. Manhattan plots corresponding to this analysis are presented in Supplementary Fig. 1, while GO terms for each additional protein category are presented in Supplementary Table 2.

**Table 1:** List of gene ontology (GO) terms related to proteins with lower and higher relative abundance in sperm from subfertile TB stallions when compared to sperm from fertile TB stallions.

ID	Source (GO term)	Term ID	Term name	*p*-value (adjusted)
*Proteins of lower relative abundance in sperm from subfertile TB stallions*				
1	CC	GO: 0,005,783	Endoplasmic reticulum	2.639 × 10^−7^
2	CC	GO: 0,012,505	Endomembrane system	1.085 × 10^−5^
3	CC	GO: 0,005,789	Endoplasmic reticulum membrane	3.044 × 10^−5^
4	CC	GO: 0,031,090	Organelle membrane	7.072 × 10^−5^
5	CC	GO: 0,005,737	Cytoplasm	3.819 × 10^−4^
6	CC	GO: 0,031,984	Organelle subcompartment	1.424 × 10^−3^
7	CC	GO: 0,031,301	Integral component of organelle membrane	3.086 × 10^−3^
11	BP	GO: 0,044,281	Small molecule metabolic process	9.674 × 10^−6^
12	BP	GO: 0,044,283	Small molecule biosynthetic process	2.660 × 10^−4^
13	BP	GO: 0,006,629	Lipid metabolic process	1.214 × 10^−3^
14	BP	GO: 0,016,126	Sterol biosynthetic process	4.675 × 10^−3^
15	BP	GO: 0,044,255	Cellular lipid metabolic process	3.869 × 10^−2^
16	BP	GO: 0,006,694	Steroid biosynthetic process	4.374 × 10^−2^
17	KEGG	KEGG: 00,071	Fatty acid degradation	5.743 × 10^−3^
18	KEGG	KEGG: 01100	Metabolic pathways	1.016 × 10^−7^
19	KEGG	KEGG: 00100	Steroid biosynthesis	1.162 × 10^−2^
20	KEGG	KEGG: 01,212	Fatty acid metabolism	1.741 × 10^−2^
22	MF	GO: 0,016,491	Oxidoreductase activity	4.214 × 10^−4^
23	MF	GO: 0,003,824	Catalytic activity	4.581 × 10^−6^
24	MF	GO: 0,016,787	Hydrolase activity	3.561 × 10^−2^
*Proteins of higher relative abundance in sperm from subfertile TB stallions*				
8	CC	GO: 0,005,940	Septin ring	1.486 × 10^−2^
9	CC	GO: 0,035,686	Sperm fibrous sheath	1.486 × 10^−2^
10	CC	GO: 0,031,105	Septin complex	3.551 × 10^−2^
21	CC	GO: 0,036,126	Sperm flagellum	1.739 × 10^−2^

The proteins were queried using *Equus caballus* orthologs. The ID numbers correspond to the IDs represented in the Manhattan plots in Fig. 3. GO: Gene ontology; *CC* cellular component; *BP* biological process; *MF* molecular function; *KEGG* Kyoto encyclopedia of genes and genomes.

### Functional network analysis of lower or higher abundance proteins in sperm from subfertile TB stallions

**Figure 4:**
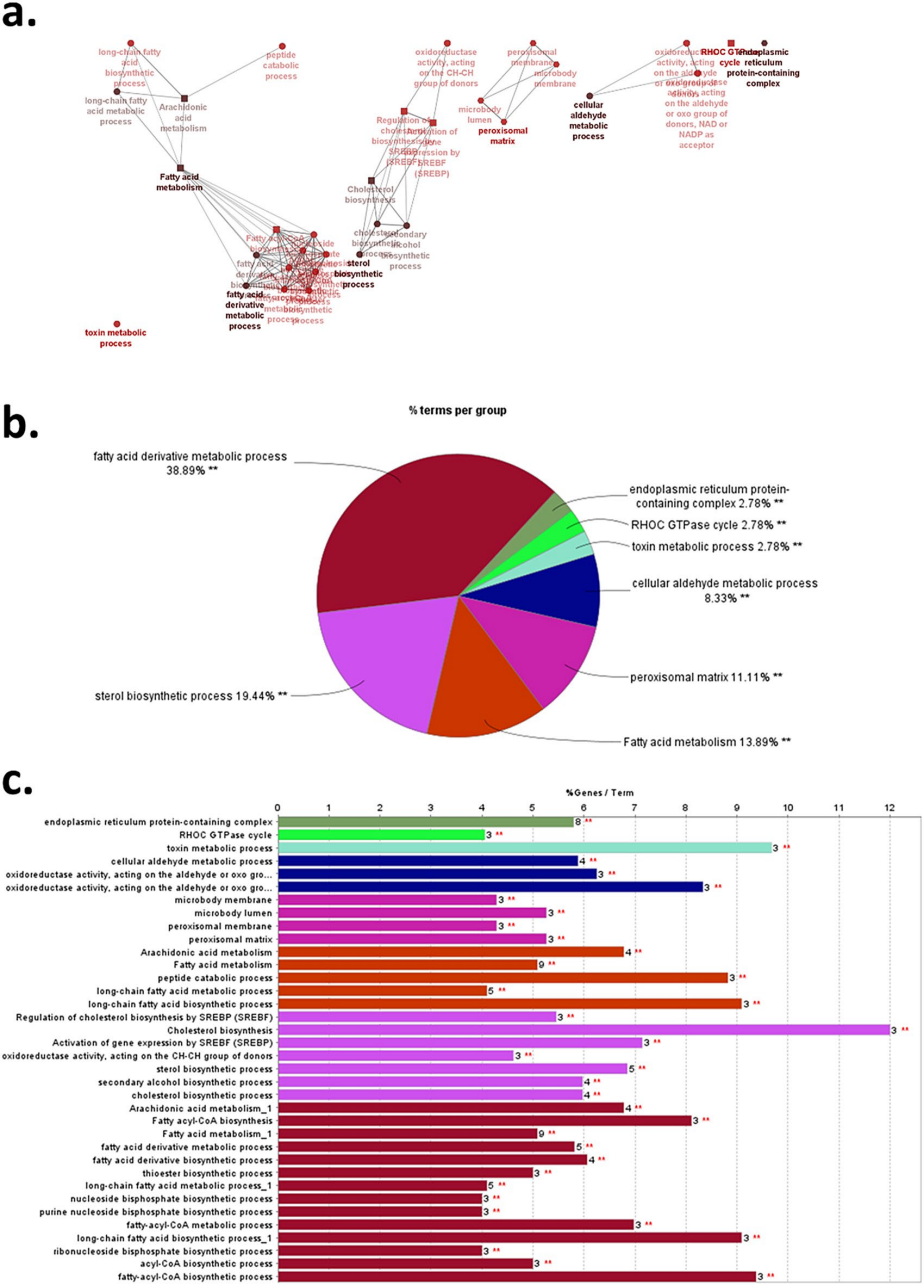
ClueGo network analysis of proteins of lower relative abundance in sperm from subfertile TB stallions. **a** GO: BP (ellipse), GO: CC (hexagon), and Reactome pathways (rectangle). Functionally grouped networks with terms are indicated as nodes based on their kappa score level (> 0.4), where only the label of the most significant term per group is shown. **b** Overview pie chart with functional groups, including specific terms for overrepresented proteins. **c** GO/pathway terms specific for overrepresented proteins. The bars represent the number of proteins associated with each term.

The ClueGo analysis tool was used in conjunction with *Homo sapiens* orthologs for GO and KEGG terms to classify the proteins of lower or higher abundance in sperm from subfertile TB stallions in a functional network. A diagram of the functional network for the proteins of lower abundance in sperm from subfertile stallions is presented in Fig. 4. The results indicated that these proteins were mainly involved in fatty acid metabolism, fatty acid derivative metabolism, sterol biosynthetic process, cellular aldehyde metabolic process, and endoplasmic reticulum protein-containing complex. Conversely, the network for the higher abundance proteins in sperm from subfertile stallions was mainly associated with vesicle docking and sperm flagellum (Supplementary Fig. 2).

### A group of proteins associated with lipid metabolism, including two enzymes of acrosomal origin, display differential abundance between stallion groups and within incubation periods

**Figure 5:**
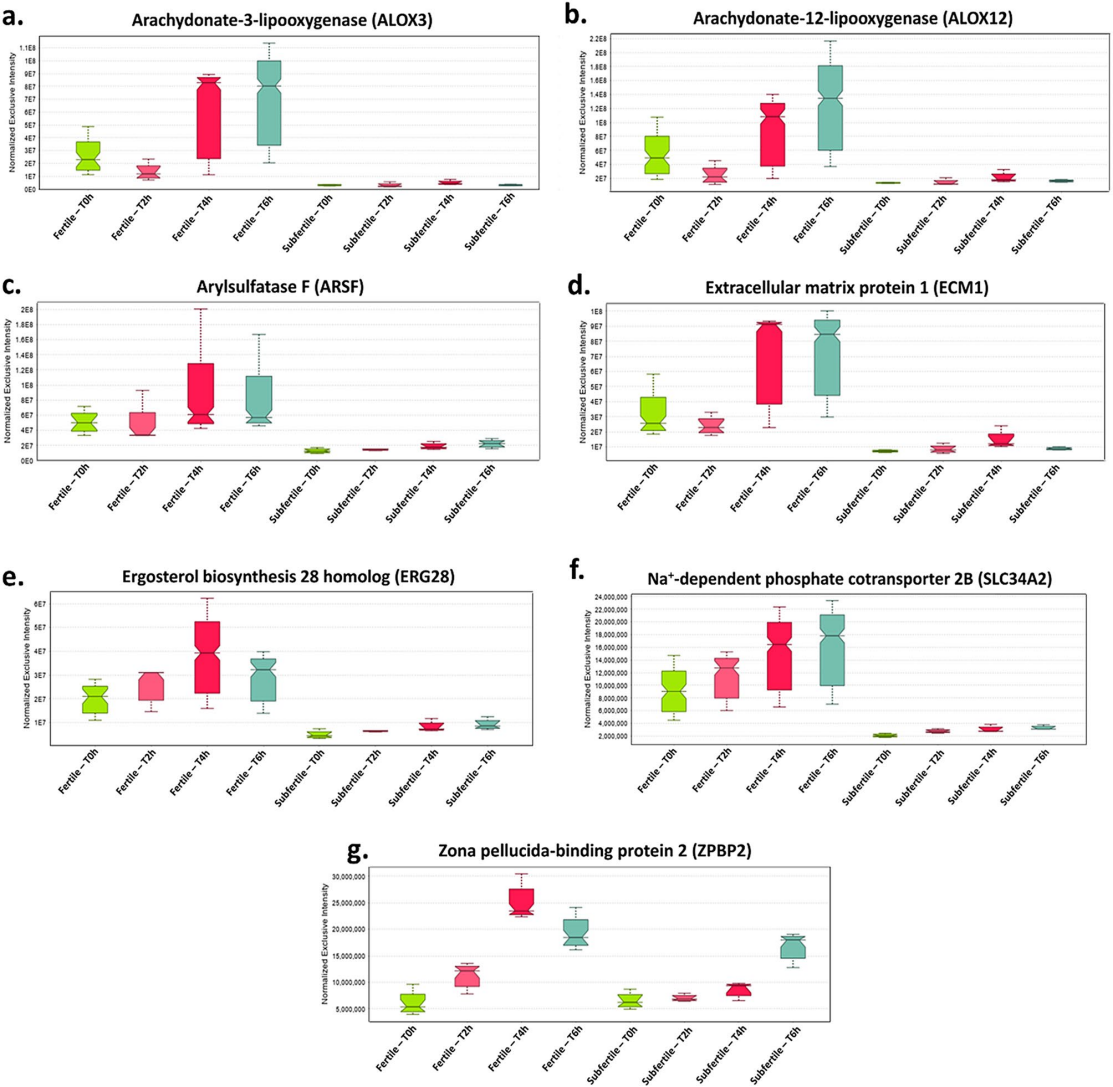
Boxplots representing the median relative abundance of the proteins arachidonate-3-lipoxygenase (ALOX3), arachidonate-12-lipoxygenase (ALOX12), arylsulfatase F (ARSF), extracellular matrix protein 1 (ECM1), ergosterol biosynthesis 28 homolog (ERG28), Na^+^-dependent phosphate cotransporter 2B (SLC34A2), and zona pellucida-binding protein 2 (ZPBP2) in sperm from fertile (left) and subfertile (right) TB stallions, in function of incubation time after exposure to Lac-MW medium.

Bioinformatic analyses suggested that most of the proteins of differential abundance, particularly those of lower abundance, in sperm from the subfertile TB stallions were associated with *metabolism* and *metabolism of lipids*. Examination of the data in Scaffold DIA was utilized to identify time-point-related changes in the relative abundance of proteins belonging to these categories. These changes were also analyzed when AE was observed in sperm from fertile but not from subfertile TB stallions (i.e., 4 and 6 h of incubation in Lac-MW medium). Proteins that fulfilled these criteria included: arachidonate lipoxygenase 3 (ALOX3), arachidonate 12-lipoxygenase (ALOX12), arylsulfatase F (ARSF), extracellular matrix protein 1 (ECM1), ergosterol biosynthesis 28 homolog (ERG28), Na^+^-dependent phosphate cotransporter 2B (SLC34A2), and zona pellucida-binding protein 2 (ZPBP2). The median relative abundance levels of these proteins between stallion groups and within periods are presented in Fig. 5.

### The expression of the acrosome protein arylsulfatase F (ARSF) in sperm from fertile and subfertile TB stallions

**Figure 6:**
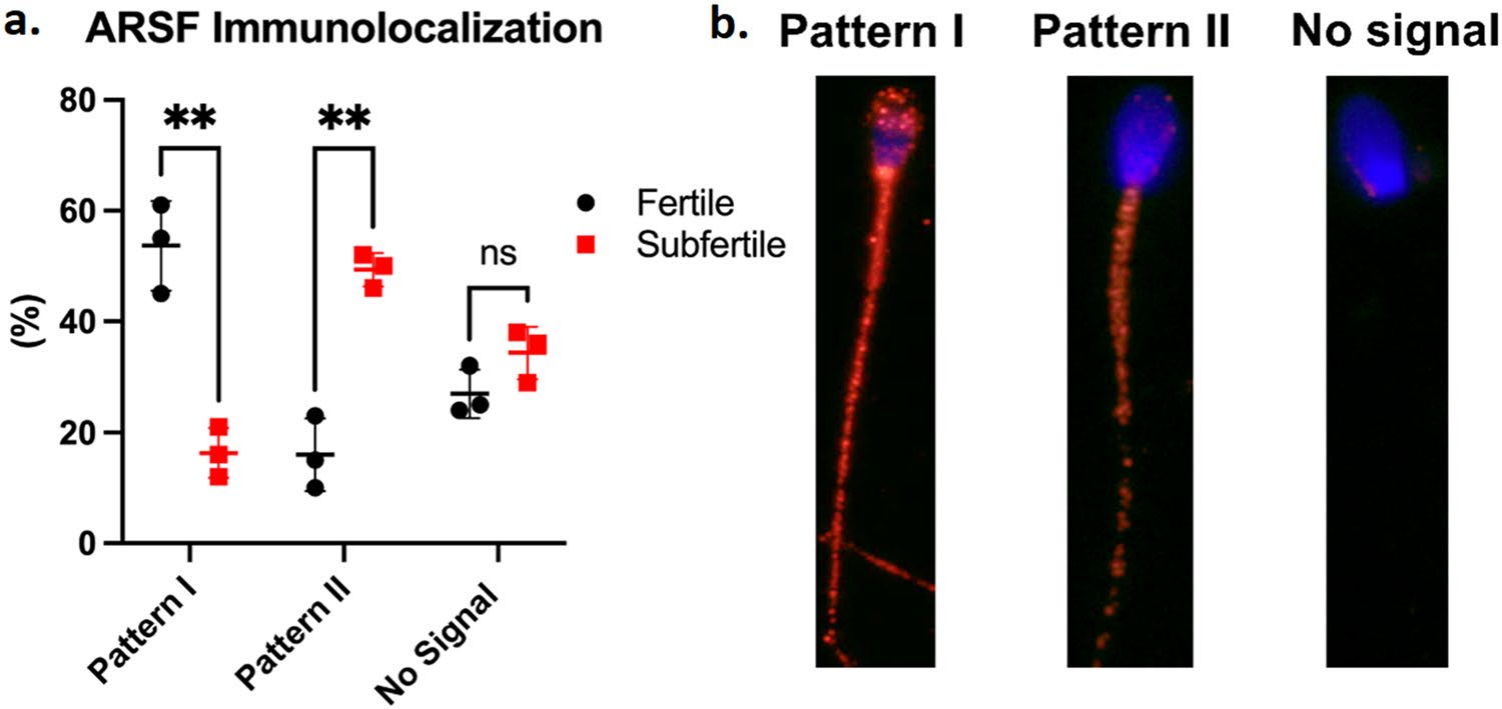
**a** The mean (± SD) percentage of frozen/thawed sperm from three fertile (n = 3) TB stallions (black dots) and three subfertile (n = 3) TB stallions (red squares) that displayed two different immunofluorescence patterns of arylsulfatase F (ARSF) localization within the sperm. Pattern I: Immunofluorescence signal at the acrosome, midpiece, and principal piece; Pattern II: Immunofluorescence signal at the midpiece and principal piece. A rabbit anti-ARSF polyclonal antibody was conjugated with a goat anti-rabbit IgG Alexa-555-secondary antibody, while the sperm nucleus was counterstained with DAPI. **b** Representative images of the immunolocalization patterns of arylsulfatase F (ARSF) in frozen/thawed stallion sperm are presented. Original magnification: 1563X. **Within immunofluorescence patterns, significant differences are indicated between stallion groups (fertile vs. subfertile).

One of the proteins of lower abundance in sperm from subfertile TB stallions is an enzyme of acrosome origin and might have relevance during the sperm-oocyte interaction process. As such, we sought to determine if this protein was present in stallion sperm and whether its expression and location differed between sperm of fertile and subfertile TB stallions. By indirect immunofluorescence, we observed that the protein arylsulfatase F (ARSF) was expressed at a higher proportion at the acrosome, mid-, and principal piece in sperm (Pattern I) from fertile TB stallions than in sperm from subfertile TB stallions (mean ± SD: 57 ± 8% vs. 17 ± 5%, respectively; *P* < 0.05), while at a lower proportion at the midpiece and principal piece (Pattern II) in sperm from fertile TB stallions than in sperm from subfertile TB stallions (mean ± SD: 16 ± 7% vs. 49 ± 3%, respectively; *P* < 0.05). Representative immunofluorescence images of the location of ARSF in stallion sperm are presented in Fig. 6.

### Frozen/thawed sperm from subfertile TB stallions display a lower ability to bind to the zona pellucida than frozen/thawed sperm from fertile TB stallions

**Figure 7:**
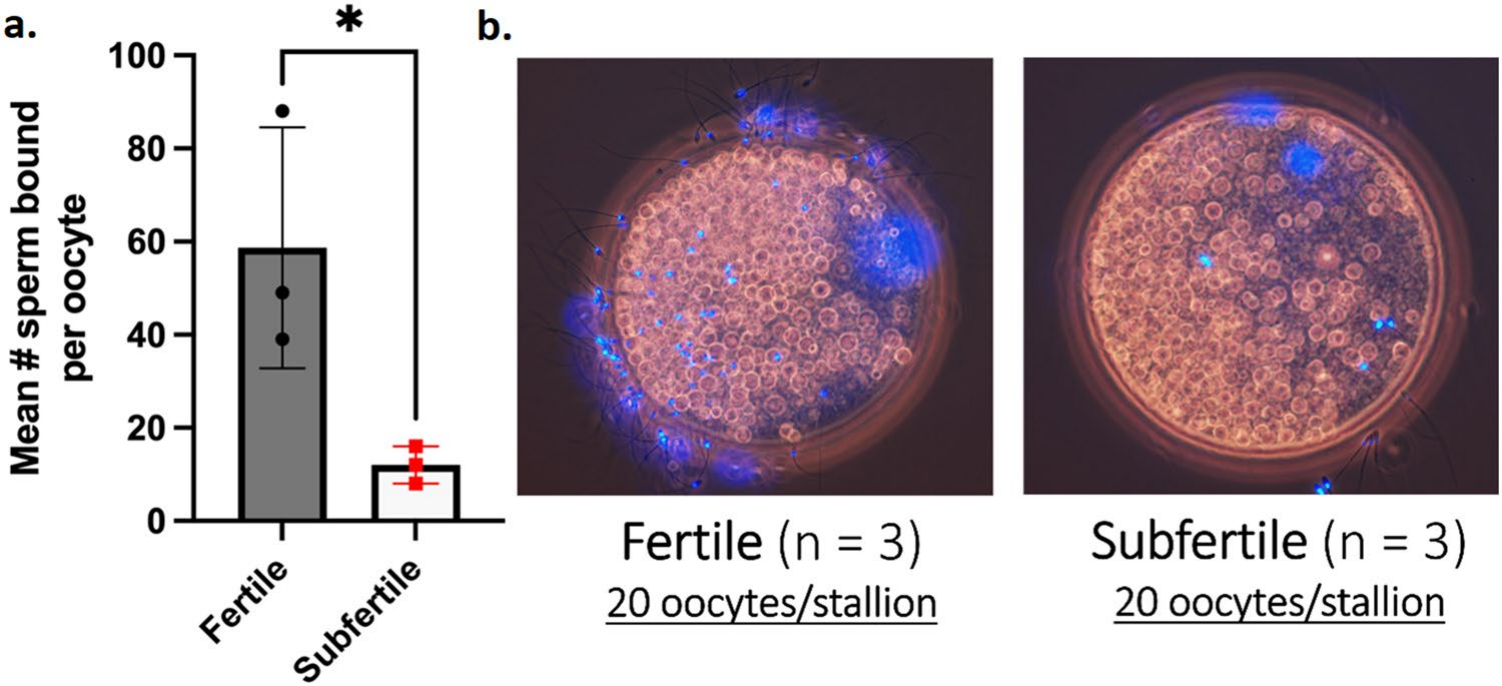
**a** The mean number of frozen/thawed sperm from three fertile (n = 3) TB stallions (dark grey bar) or three subfertile (n = 3) TB stallions (light grey bar) that bound to porcine zona pellucida following incubation in Lac-MW for 4 h. **b** Representative microphotographs obtained by phase-contrast and fluorescence microscopy of an oocyte inseminated with sperm from a fertile stallion (left) and a subfertile stallion (right) are presented (original magnification: 400X). * Indicates significant differences between groups (fertile vs. subfertile) in the mean number of sperm bound to the zona pellucida.

A total of 120 in vitro-matured porcine oocytes (20 oocytes per stallion, 60 for fertile TB, and 60 for subfertile TB stallions, respectively) were used in this experiment. The mean number of sperm bound to the ZP was higher in fertile TB than in subfertile TB stallions (55 ± 8 sperm/ZP vs. 15 ± 4 sperm/ZP; *P* < 0.05; Fig. 7).

## Discussion

In the current study, we identified candidate proteins further to investigate the causes of IAE in TB stallions. For this purpose, we combined the use of a method to induce AE in viable sperm (i.e., incubation in a lactate-only containing medium [Lac-MW]) and DIA-MS to identify and quantify the relative levels of proteins in the sperm of both fertile and subfertile TB stallions. Previous studies have used mass spectrometry-based approaches to investigate the stallion sperm proteome to identify potential proteins associated with higher sperm quality or fertility potential^15,22,36,37^, or to determine the effects of storage methods (i.e., cooled storage or freezing/thawing) on the sperm proteome^16,38–41^. To the best of our knowledge, the current study is the first to use this technology to investigate the potential cause(s) of a clinical condition in stallions that is unique due to the phenotype that these individuals present: reduced in vivo fertility associated with IAE despite having normal-to-excellent conventional sperm quality parameters.

Our results indicate that most of the proteins of differential abundance in sperm from the subfertile TB stallions correspond to processes associated with either cell metabolism or metabolism of lipids, as observed in the gene ontology analyses using both *Equus caballus* or *Homo sapiens* orthologs. These results are interesting from two perspectives: 1) both sperm capacitation and AE require extensive remodeling of the sperm plasma membrane to prepare the fusion between both plasma and outer acrosomal membranes, facilitating the release of acrosomal contents^42–46^. This remodeling process, in turn, requires cholesterol depletion from the plasma membrane, allowing calcium entry at the intracellular level and activating second messenger pathways^47–50^. Some of the biochemical changes related to sperm capacitation and AE are also associated with the metabolism of lipids at the sperm membrane, particularly by the activation of phospholipase A2 (PLA2), which will result in the production of arachidonic acid metabolites and lysophospholipids that enhance the initiation of AE^51–55^. Furthermore, both sperm capacitation and AE require considerable quantities of energy in the form of ATP and are also governed by a delicate balance between the consumption of energy and the production of reactive oxygen species (ROS)^56–58^; thus, it would be expected that proteins associated with metabolic processes would be overrepresented in proteomic analysis of sperm.

We did not observe an overall interaction between the stallion condition (fertile vs. subfertile) and the sperm AE during incubation periods using the Lac-MW medium. There may be differences in protein post-translational modifications (PTMs) across the various time points. Since biological PTMs were not evaluated in our DIA-MS analyses, no conclusions can be reached regarding this study. We were able to identify changes over time in seven proteins (ALOX3, ALOX12, ARSF, ECM1, ERG28, SLC34A2, ZPBP2) that were differentially abundant when sperm from fertile stallions underwent AE (i.e., T4h and T6h). These proteins also were of lesser abundance in sperm from the subfertile stallions when compared to fertile stallions (Fig. 5). Gene ontology analysis using the g:Profiler server revealed that these proteins corresponded to the terms *metabolism* and *metabolism of lipids*. These findings are consistent with an earlier study from our group indicating that subfertile TB stallions that carry the susceptibility genotype for IAE have a higher cholesterol-to-phospholipid ratio in their sperm membranes and seminal plasma^59^. As such, it is possible that the acrosomal dysfunction observed in these individuals could be related to excessive levels of cholesterol in the sperm membrane that influence the ability of sperm to undergo either capacitation or AE^59^. The changes in the relative abundance of these proteins also coincided with the maximum levels of AE in viable sperm, as determined by flow cytometry. Two of these proteins, ALOX3 and ALOX12, correspond to enzymes associated with the metabolism of fatty acids into leukotrienes, which is related to an increase in the lipid peroxidation levels of biological membranes^60^. Contrasting results have indicated that lipoxygenases may^61,62^, or may not^63^ be associated with AE in hamster, bull, or human sperm incubated under capacitating conditions. A recent study showed that inhibition of ALOX15 in human sperm resulted in increases in sperm motility, calcium ionophore A23187-induced AE, and sperm-ZP binding compared to samples that were only treated to stimulate oxidative stress but in which ALOX15 was not inhibited^64^. Yet, these results should not be interpreted that arachidonate lipoxygenases induce only deleterious effects on sperm, but rather that a balance between the pro-oxidative effects of these lipoxygenases must exist to induce redox changes associated with sperm capacitation and AE, and not only associated with oxidative stress and cell death. Another protein of interest is ERG28. Studies in human sperm indicate that the addition of ergosterol to sperm incubated under capacitating conditions for 24 h resulted in a reduced rate of spontaneous and progesterone-induced AE^65^. In that study, the reduced rate of AE caused by the addition of ergosterol to sperm was related to its similar effects on the cholesterol-to-phospholipid ratio on the sperm membranes, as ergosterol is structurally similar to cholesterol and can be incorporated within the sperm plasma membrane similarly as cholesterol^65^. This contrasts with our results, which show a higher relative abundance of ERG28 in the sperm from fertile stallions with a higher rate of spontaneous AE in viable sperm. RT-PCR analysis indicated that ERG28 is highly expressed in human testicular tissue^66^, and the role of this protein in *Saccharomyces cerevisiae* suggests that this protein is necessary for the normal progression of the ergosterol biosynthetic pathway^67^. However, the actual localization of ERG28 within the testicular cell types or its potential relevance in male reproductive physiology has not yet been described. The role of a Na^+^-dependent phosphate cotransporter (SLC34A2) during sperm capacitation or AE could be explained by the requirements of membrane hyperpolarization that lead to the entry of calcium within the sperm and the increase of intracellular pH^68,69^; nonetheless, this specific cotransporter has not been previously identified in sperm from any species.

Arylsulfatase, particularly arylsulfatase A (ARSA), has been identified in sperm from rabbits, mice, and humans in the post-acrosomal region and plasma membrane^70–72^. This protein remains at the sperm post-acrosomal region after AE^70,71^ and is implicated in sperm-oocyte binding due to its activity as a sulfatase that interacts with the sulfoglycoproteins present in the receptors ZP2 and ZP3^73–75^. In the present study, another member of the arylsulfatase gene family, arylsulfatase F (ARSF), was observed by immunofluorescence at three regions of the sperm from fertile stallions: acrosome, midpiece, and principal piece (Fig. 6). In contrast, in sperm from subfertile TB stallions, the ARSF signal was only observed at the sperm midpiece and principal piece (Fig. 6). Such marked difference in the localization of ARSF at the acrosome region might explain the reduced ability of sperm from subfertile stallions to both undergo AE and bind to the ZP. Further studies in which the immunolocalization of ARSF in stallion sperm is studied after incubation under capacitating conditions and after the occurrence of AE are warranted.

Another protein of interest identified by DIA-MS in our study was zona pellucida-binding protein 2 (ZPBP2), which has been identified in proacrosomal vesicles that later integrate the inner acrosomal membrane in sperm from other species^76^. This protein is not only released from sperm during AE, acting as a secondary receptor for sperm-ZP binding^77^, but also interacts with other proteins such as testisin, which recently has been identified in stallion sperm as an essential serine protease required for normal sperm capacitation, AE, and ZP-binding^78^. Interestingly, in a recent case report involving a TB stallion with considerably low in vivo fertility, zona pellucida-binding protein was identified as one potential candidate biomarker for impaired acrosomal exocytosis, based on mass spectrometry-based analysis^21^. The stallion involved in that case report had a reduced rate of AE following stimulation with calcium ionophore A23187, a similar finding to what we have previously observed in TB stallions that carry the IAE susceptibility genotype^5,7^. Unfortunately, the case report by Swegen et al.^21^ did not indicate whether the affected stallion also carried the IAE susceptibility genotype. Nonetheless, according to the clinical phenotype, it seems plausible that the TB stallion from that report had the identical IAE susceptibility haplotypes as the stallions used in the current study; as such, the zona pellucida-binding protein can be considered an important marker protein for IAE in TB stallions. While in our study we did not identify by immunofluorescence the presence of ZPBP2 in sperm from either fertile or subfertile TB stallions, a potential confirmation of reduced zona pellucida-binding protein function in sperm from subfertile TB stallions was their reduced ability to bind to porcine ZP after incubation under capacitating conditions (Fig. 7).

In the present study, we utilized frozen/thawed sperm from fertile and subfertile TB stallions, mainly due to the inability to access fresh semen from these individuals, as mentioned above. Freezing and thawing alter the proteome of stallion sperm, resulting in a lower abundance of several proteins involved in metabolism regulation and redox regulation^38,40,41^. Proteins related to sperm-oocyte interactions, namely IZUMO-4 and zona pellucida binding protein, had lower abundance in frozen/thawed stallion sperm when compared to freshly ejaculated sperm^38^. In the current study, we did not observe statistical differences in the relative abundance of any of the IZUMO proteins (1, 2, 3, or 4) between stallion groups or within periods (data not shown), and we only detected differences in the relative abundance of zona pellucida-binding protein, as described above. Since we did not compare the proteome of fresh versus frozen/thawed sperm from the stallions enrolled in the present study, we cannot compare our results with those presented by Martin-Cano et al.^38^.

Notably and despite the compelling evidence from several studies that the double homozygous A/A-A/A genotype in *FKBP6* exon 5 is significantly associated with the occurrence of IAE in TB stallions^5,7,8^, we did not detect the presence of the FKBP6 protein in sperm from any of the stallion groups tested. This is consistent with the recent theory that *FKBP6* is not the causative gene for IAE, and the A/A-A/A genotype in exon 5 is rather tagging a haplotype unique to Thoroughbreds with IAE^8^. The current results might offer insight into the mechanisms underlying IAE given the reported high cholesterol-to-phospholipid ratio in the sperm membranes^59^ and the reduced acrosomal function after non-physiological^4,5^, or more physiological stimulation of AE^6^. Future investigations will be focused on identifying the dynamics (i.e., localization or patterns of expression) of some of these proteins during the initiation of capacitation and AE in stallion sperm.

## Conclusions

The current study explored the sperm proteome in fertile and subfertile TB stallions, with the latter being carriers of the susceptibility genotype for IAE. Using a DIA-MS approach and a method that induces AE in viable sperm, we identified a group of differentially abundant proteins associated with metabolism and metabolism of lipids that may explain the acrosomal dysfunction observed in subfertile stallions. One of these proteins, ARSF, was detected at the acrosome, midpiece, and principal piece in sperm from fertile TB stallions but only at the midpiece and principal piece in sperm from subfertile TB stallions. We also provide evidence that the ability of frozen/thawed sperm from subfertile TB stallions to bind to porcine ZP was decreased compared to that of fertile TB stallions. Some of the proteins identified by mass spectrometry and immunofluorescence in the current study are candidates for further studies focused on determining the pathophysiological cause of IAE and understanding the biological processes involved in sperm capacitation and AE in stallions.

## Materials and methods

## Ethics statement

All animal studies were conducted in compliance with the Texas A&M University Institutional Animal Care and Use Committee (IACUC 2021–0007) and followed the ARRIVE guidelines for ethical research involving live animals.

### Reagents and media

Unless otherwise stated, reagents were purchased from Millipore Sigma (St. Louis, MO, USA). Fixable Live/Dead Red Stain, rabbit anti-arylsulfatase F (ARSF) polyclonal antibody, goat anti-rabbit IgG Alexa-555-conjugated secondary antibody, 10% normal goat serum, SlowFade™ Diamond Antifade Mounting Solution with DAPI, and methanol-free 16% paraformaldehyde were acquired from Thermo Fisher Scientific (Waltham, MA, USA). Accumax^®^ Cell Detachment Solution was purchased from Stemcell™ Technologies Inc. (Cambridge, MA, USA). Acridine Orange stain was obtained from Polysciences Inc. (Warrington, PA, USA). CryoMax Lactose-EDTA® semen freezing extender (20% egg-yolk + 2% glycerol and 3% methyl formamide) was obtained from Animal Reproduction Systems (Chino, CA, USA). A silane-coated silica particle solution (Redigrad®) for density gradient centrifugation was acquired from Global Life Sciences Solutions (Marlborough, MA, USA). The base medium, MW-HEPES, used for sperm washing by density gradient centrifugation was a modified Whitten’s medium^79^ and consisted of 110 mM NaCl, 4.7 mM KCl, 1.2 mM MgCl_2_, 1.9 mM CaCl_2_, 22 mM HEPES, and 50 µL/mL gentamicin sulfate. The medium used for sperm in vitro incubation under capacitating conditions (Lac-MW) was prepared as reported previously^6,79^ and consisted of modified Whitten’s medium with 25 mM HCO_3_^−^ instead of HEPES, 7 mg/mL bovine serum albumin—heat shock fraction (BSA), and 10 mM sodium-DL lactate (60% syrup). All media were adjusted with NaCl to an osmolality of 280–290 mOsm/kg. On the day of the experiment, the pH of each medium was adjusted to 7.25 using NaOH or HCl. The Lac-MW medium was maintained at 38.2 °C in 5% CO_2_ for at least 2 h before use.

### Stallions and semen collection

All stallions enrolled (n = 6) were Thoroughbred, sexually active, and 7–15 years old. Hair samples were procured to determine the presence of the susceptibility genotype for IAE, A/A-A/A in the gene *FKBP6* exon 5, as previously reported^7,8^. The *FKBP6* genotype, in vivo fertility rates, and conventional sperm quality parameters of the six stallions used (three fertile and three subfertile) are presented in Supplementary Table 1. Before semen collection, each stallion was exposed to an ovariectomized mare (when available) or a mare in standing estrus. Once erect, the penis was rinsed thoroughly with warm water and dried with paper towels. The ejaculates from two fertile TB stallions and one subfertile TB stallion were collected using a Colorado-type artificial vagina (Animal Reproduction Systems, Chino, CA, USA), while ejaculates from the other three TB stallions (one fertile and two subfertile) were collected using a Missouri-Model artificial vagina (Nasco, Ft. Atkinson, WI, USA). For both artificial vagina types, an in-line nylon micromesh filter (Animal Reproduction Systems) was placed between the artificial vagina and the semen collection receptacle to separate the gel fraction from the gel-free semen. Following semen collection, the gel-free semen was transported to an adjacent laboratory and placed in an incubator (37 °C) before processing.

### Initial semen processing and analyses

The gel-free semen volume was estimated based on sample weight, while sperm concentration and plasma membrane intactness (i.e., viability) were measured using a fluorescence-based cell counter (NucleoCounter SP-100™, Chemometec A/S, Allerød, Denmark), following a previously described methodology^80^. Sperm motion characteristics were determined using computer-assisted sperm analysis (CASA; Hamilton-Thorne IVOS II, Hamilton-Thorne Inc., Beverly, MA, USA), as reported previously^81^. The preset values for the instrument consisted of the following: frames acquired, 45; frame rate, 60 Hz; minimum contrast, 70; minimum cell size, four pixels; minimum static contrast, 30; straightness (STR) threshold for progressive motility, 50%; average path velocity (VAP) threshold for progressive motility, 30 µm/s; VAP threshold for static cells, 15 μm/s; cell intensity, 106 pixels; static head size, 0.60 to 2.00 μm; static head intensity, 0.20 to 2.01; static elongation, 40 to 85; illumination intensity, 2200. Sperm motility parameters included the percent of total motility (TMOT), progressive motility (PMOT), and the mean curvilinear velocity (µm/s; VCL). For sperm morphology analysis, samples of raw semen were fixed with buffered-formal saline (BFS; 4.75% formaldehyde) and analyzed using differential interference contrast (DIC) microscopy (1563x, Olympus BX-60, Olympus Corporation, Melville, NY, USA). Sperm morphological classification was done as previously reported^82^. All morphologic abnormalities were counted for each spermatozoon to determine the incidence rate. A total of 100 sperm were counted for each ejaculate, and the percentage of morphologically normal sperm was recorded. Sperm DNA quality was determined in flash-frozen/thawed samples obtained from raw semen using the Sperm Chromatin Structure Assay (SCSA), as previously described^83^. The percentage of Cells Outside the Main Population (COMP_α-t_), also known as DNA fragmentation index (DFI), was used as an endpoint to determine the extent of the susceptibility of sperm DNA to denaturation.

### Semen cryopreservation and stimulation of lactate-induced AE in viable frozen/thawed sperm

In a previous study, we determined that stallion sperm stored at 5 °C for 24 h or frozen/thawed stallion sperm undergo spontaneous AE in viable sperm at the same rate as fresh semen after being incubated in a Lac-MW medium^6^. Because all the subfertile TB stallions that our group has identified as carrying the IAE susceptibility genotype were either located far from our laboratory, had been already castrated, or their sperm were cryopreserved several years previously, in the current study, we used frozen/thawed sperm from these stallions to perform the acrosome function and subsequent DIA-MS analyses. When these ejaculates were obtained, immediately after semen collection and initial sperm analysis, the raw semen was diluted 1:1 (v/v) with INRA-96^®^ extender (IMV Technologies, L’Aigle, France) and subjected to cushioned centrifugation at 1000 × *g* for 20 min, as described previously^84^. After centrifugation, the supernatant was removed, and the sperm pellet was resuspended with the EZ-Freezin CryoMax LE® semen freezing extender (Animal Reproduction Systems, Chino, CA) at a final sperm concentration of 200 × 10^6^ sperm/mL. Sperm diluted with the freezing extender was loaded into 0.5-mL plastic straws, sealed ultrasonically, and frozen in a controlled rate freezer (CBS 2100; Custom Biogenic Systems, Bruce Township, MI, USA) using the following cooling curve: − 2.0 °C/min from 25 to 20 °C; − 0.1 °C/min from 20 to 5 °C; hold for 5 min; − 60 °C/min from 4 to − 140 °C^85^. The straws were plunged directly into liquid nitrogen and stored in a liquid nitrogen tank. Frozen straws from each stallion were thawed for 30 s in a water bath set at 37 °C and the thawed semen was processed through density gradient centrifugation using 40% Redigrad® (Global Life Science Solutions, Marlborough, MA^86^) to remove seminal plasma, debris, and semen extender. After centrifugation, the sperm pellet was diluted to 30 × 10^6^ sperm/mL in Lac-MW medium^6,79^ and incubated for 6 h at 38.2 °C in 5% CO_2_. Sperm aliquots were analyzed after 0, 2, 4, and 6 h of incubation (T0h, T2h, T4h, and T6h, respectively) in Lac-MW medium for viability/acrosomal exocytosis (AE-Viable), as previously reported^6^. At each time point (T0h, T2h, T4h, and T6h), a 30 × 10^6^ sperm aliquot was also flash-frozen in dry ice for further proteomic analysis using DIA-MS.

### *Analysis of sperm viability/acrosomal exocytosis (Fixable Live/Dead Red stain* + *FITC-PSA)*

The intactness of the plasma membranes (viability) and the acrosome membranes (AE) was evaluated simultaneously, as previously described^87^, with some modifications. An aliquot (50 µL) of frozen/thawed semen diluted (30 × 10^6^ sperm/mL) in Lac-MW was added to 1 mL of Lac-MW medium. This dilution resulted in a final sperm concentration of approximately 1.5 × 10^6^ sperm/mL. One µL of Fixable Live/Dead Red stain (Excitation: 488 nm, Emission: 617 nm; final concentration: 50 µg/mL) was added to the sperm sample and incubated at 38.2 °C in an air atmosphere for 20 min. Then, 140 µL of methanol-free paraformaldehyde (paraformaldehyde final concentration: 1.88% v/v) was added, and the sperm sample was stored at 5 °C in the dark for 30 min. Subsequently, the samples were centrifuged (400 × *g* × 5 min) using BSA in DPBS (2 mg/mL), permeabilized with Triton-X100 (1% v: v), centrifuged again with BSA in DPBS, diluted in 133 µL Accumax to avoid sperm clumping, and incubated with 10 µL *Pisum sativum* agglutinin (PSA)-FITC conjugate (excitation, 488 nm; emission, 517 nm; final concentration, 0.0375 mg/mL) for 20 min at room temperature in the dark. The Fixable Live/Dead Red stain binds to free amines both in the intracellular space and the surface of cells with a disrupted plasma membrane (“non-viable” cells)^88^. In the case of the Fixable Live/Dead Red stain, non-viable sperm will allow the internalization of this dye and emit red fluorescence^87^. PSA binds to the glycoconjugates of the acrosomal matrix, particularly to the α-D-glucosyl and the α-D-mannosyl residues at the inner acrosomal membrane^89^. In non-fixed/non-permeabilized sperm, when the acrosomal membrane is disrupted due to acrosome damage or during AE, FITC-PSA will bind to such residues and emit a green, fluorescent signal. On the contrary, in both fixed and permeabilized sperm, the FITC-PSA stain will bind to non-acrosome disrupted sperm and emit green fluorescence. In contrast, upon acrosomal disruption, this fluorescent signal will be lost. Hence, four subpopulations are identified using Fixable Live/Dead Red stain and FITC-PSA: 1) sperm with both intact plasma and acrosomal membranes [FITC-PSA ( +)/Fixable Live/Dead Red ( −)]; 2) sperm with intact plasma membrane and disrupted acrosomal membrane [FITC-PSA ( −)/Fixable Live/Dead Red ( −)]; 3) sperm with damaged plasma membrane and intact acrosomal membrane [FITC-PSA ( +)/Fixable Live/Dead Red ( +)]; 4) sperm with both damaged plasma and acrosomal membranes [FITC-PSA ( −)/Fixable Live/Dead Red ( +)]. Following incubation with FITC-PSA, the samples were diluted in 150 µL Accumax and processed immediately using a flow cytometer (FACScan, Beckton Dickinson, Mountain View, CA) equipped with a 488-nm argon laser at 20 mW and three fluorescent detectors (FL1, bandpass 530/30 nm; FL2, bandpass 585/42 nm; and FL3, long pass 670 nm). The voltage settings on the flow cytometer were: FSC-H, 553; SSC, 240; FL1, 741; and FL2, 821. The compensation was set on FL2 as 98% of FL1. The FITC-PSA signal was acquired using the FL1 filter, while the Fixable Live/Dead Red stain signal was acquired using the FL2 filter. The flow rate was 200–400 sperm/s, and a minimum of 5000 sperm were analyzed per sample. To identify doublets and clumps that could affect the analysis and interpretation of sperm events, a manual gating strategy was applied whereby the FSC and SSC were plotted. This methodology has been validated in previous reports from our laboratory^6,79^. Flow cytometry data were analyzed using WinList™ software (Verity Software House, Topsham, ME, USA). The percentage of AE in viable sperm (AE/Viable) was considered as the experimental endpoint. This parameter is calculated by dividing the percentage of AE in viable sperm (bottom left quadrant in the scatterplot) by the percentage of viable sperm in the sample (bottom left + bottom right quadrants in the scatterplot)^6^.

### Sperm preparation for protein extraction

Aliquots of frozen/thawed sperm (30 × 10^6^ sperm) incubated at either 0 h, 2 h, 4 h, or 6 h in Lac-MW medium were flash/frozen in dry ice until further processing and then thawed for 1 min at 37 °C in a water bath. Immediately after thawing, 10 µL of EDTA-free protease inhibitor cocktail (Pierce™ Protease Inhibitor Tablets, Thermo Fisher Scientific, Waltham, MA) was added per each sperm aliquot. The sperm were washed two times in calcium-free PBS at 400 × *g* for 5 min, pelleted, maintained frozen at − 80 °C, and sent to the Institutional Mass Spectrometry Core Laboratory at the University of Texas Health Science Center at San Antonio (San Antonio, Texas) for further analysis.

### Protein identification and relative quantification by data-independent acquisition mass spectrometry (DIA-MS)

Protein aliquots corresponding to 70 µg protein, as quantified using the EZQ™ Protein Quantitation Kit (Thermo Scientific, Waltman, MA), were reduced with tris(2-carboxyethyl)phosphine hydrochloride (TCEP-HCl; Pierce™, Thermo Fisher Scientific), alkylated in the dark with iodoacetamide and applied to S-Traps spin columns (Protifi™, Farmingdale, NY) for tryptic digestion (sequencing grade; Promega) in 50 mM triethylammonium bicarbonate (TEAB; Thermo Fisher Scientific). Peptides were eluted from the S-Traps spin columns with 0.2% formic acid in 50% aqueous acetonitrile and quantified using the Pierce™ Quantitative Fluorometric Peptide Assay (Thermo Fisher Scientific). Data-independent acquisition mass spectrometry (DIA-MS) was conducted on an Orbitrap Fusion Lumos mass spectrometer (Thermo Fisher Scientific). On-line HPLC separation was accomplished with an RSLC NANO HPLC system (Thermo Fisher Scientific/Dionex) and a PicoFrit™ nanospray column (75 µm i.d.; New Objective, Littleton, MA) packed to 15 cm with C18 adsorbent column (218MS 5 µm, 300 Å; Vydac^®^, W.R. Grace & Co., Columbia, MA); mobile phase A, 0.5% acetic acid (Hac)/0.005% trifluoroacetic acid (TFA; Pierce™, Thermo Fisher Scientific) in water; mobile phase B, 90% acetonitrile/0.5% Hac/0.005% TFA/9.5% water; gradient 3 to 42% B in 120 min; flow rate: 0.4 µL/min. A pool was made of all samples, and 2 µg aliquots of the digests were analyzed using gas-phase fractionation and 4-*m/z* windows [three mass ranges (395–605mz, 595–805mz, 795–1005mz), staggered; 30 k resolution for precursor and product ion scans, all in the orbitrap] to create a DIA chromatogram library^90^ by searching against a Prosit-generated predicted spectral library^91^ based on the UniProt *Equus caballus* protein sequence database (44,488 sequences, downloaded on 12–11-2020): peptide mass tolerance, 10.0 ppm; fragment mass tolerance, 10.0 ppm; fixed modification, carbamidomethylation (C); enzyme, trypsin with a maximum of one missed cleavage; peptide charge state, + 2– + 3; peptide length, 6–30; protein FDR, 1%; minimum of two identified peptides; peptide quantification, Encyclopedia (0.8.1) based on the five highest quality fragment ions. Experimental samples were blocked by replicate and randomized within each replicate for sample preparation and analysis, employing 2 µg of peptides and the 2-h HPLC gradient described above. MS data for experimental samples were acquired in the orbitrap using 8-*m/z* windows (390-1010mz, staggered; 30 k resolution for precursor and product ion scans) and searched against the chromatogram library using the same parameters as described above for generation of the chromatogram library. Scaffold DIA (v3.2.1; Proteome Software) was used for all DIA-MS data processing. The data files were converted to mzML format using ProteoWizard (3.0.19254)^92^. Deconvolution of the staggered windows was performed. Peptides identified in each sample were filtered by Percolator (3.01.nightly-13-655e4c7-dirty)^93^ to achieve a maximum false discovery rate (FDR) of 0.01. Individual search results were combined, and peptide identifications were assigned posterior error probabilities and filtered to an FDR threshold of 0.01 by Percolator. Peptide quantification was performed by Encyclopedia (version 1.12.31)^90^. For each peptide, the five highest-quality fragment ions were selected for quantification. Proteins that contained similar peptides and could not be differentiated based on MS/MS analysis were grouped to satisfy the principles of parsimony. Quartile normalization was applied on the log10 peptide intensities across replicates.

### Bioinformatic analyses

Scaffold DIA was used to assess differences in the results for samples from fertile and subfertile stallions (A/A-A/A) across the selected periods (T0h, T2h, T4h, or T6h). A two-way ANOVA design, available in the Scaffold DIA software, was used in which the primary category of analysis was the stallion phenotype (fertile vs. subfertile), while the second category of analysis was the time point (T0h vs. T2h vs. T4h vs. T6h). Significance was assessed using Benjamini–Hochberg multiple testing correction^94^, with a false discovery rate (FDR) q-value of 0.05, resulting in a cutoff of *P* < 0.0069. Proteins that exhibited log_2_(fold change) ≤  − 0.585 or ≥ 0.585 (i.e., fold change in protein abundance between fertile and subfertile stallions 1.5 × up or down) were used to filter the results for further analysis.

The proteins identified as differentially abundant between stallion groups and among periods were queried for gene ontology (GO) terms using g: Profiler^95^, according to cellular component (CC), biological process (BP), and molecular function (MF). The overrepresentation of differential abundance proteins was queried using *Equus caballus* orthologs and a g: SCS threshold value of 0.05. The Kyoto Encyclopedia of Genes and Genomes (KEGG) database was also used in g: Profiler to analyze the overrepresentation of biological pathways. In addition, pathway enrichment analyses were conducted utilizing both the g: Profiler and Reactome servers using *Homo sapiens* orthologs, given the increased depth of the human proteome in terms of annotation. A network analysis of the differentially abundant proteins was performed using the Cytoscape (version 3.9.1) plugin ClueGo (version 2.5.8)^96^ to identify functionally grouped GO terms.

### Detection of acrosome proteins of differential abundance between fertile and subfertile TB stallions by immunofluorescence

Indirect immunofluorescence of the protein arylsulfatase F (ARSF) was performed, as previously described^6^, with some modifications. Briefly, frozen/thawed semen from fertile and subfertile TB stallions (n = 3 stallions per group) was processed by density gradient centrifugation using 40% Redigrad and diluted to 30 × 10^6^ sperm in Lac-MW. Next, the sperm were centrifuged (600 × *g* for 5 min) three times in DPBS, applied to poly-L-lysine coated slides for 2 h at room temperature, and exposed to 4% paraformaldehyde in DPBS for 20 min at 4 °C. As ARSF is a protein of acrosome origin, we did not expose the sperm to any permeabilizing agent (i.e., triton X-100) to avoid any loss of proteins at the outer acrosomal membrane or acrosomal matrix. Following fixation, the slides were washed again three times using DPBS, blocked with 10% normal goat serum for 1 h at room temperature, and washed again three times using DPBS. A rabbit anti-ARSF, diluted 1:100 in 10% normal goat serum, was added to the slides and incubated overnight at 4 °C. A negative control was produced in which the slides were not exposed to the primary antibody (i.e., anti-ARSF). Following overnight incubation, the sets of slides were washed three times with DPBS and stained for 1 h at room temperature with a goat anti-rabbit IgG Alexa-555-conjugated secondary antibody (excitation: 555 nm; emission: 572 nm) at a 1:100 dilution in 10% normal goat serum. A final washing step was performed using DPBS; then, 3.5 µL of the SlowFade Mountant with DAPI was added to prevent fluorescence bleaching and counterstain the sperm nuclei. Then, a coverslip was carefully applied to the slide, and the slides were evaluated using an Olympus BX-60 fluorescence microscope at a 1,563 × magnification. A total of 100 sperm were analyzed per ejaculate, and the localization of ARSF within each spermatozoon was recorded and compared between fertile and subfertile TB stallions.

### Heterologous zona pellucida-binding assay

Due to the difficulties in establishing a repeatable method for conventional in vitro fertilization of equine oocytes using frozen/thawed stallion sperm, we performed a heterologous zona pellucida-binding assay to determine the ability of stallion sperm incubated in Lac-MW to undergo acrosomal exocytosis and bind to the zona pellucida (ZP)^97,98^. Porcine oocytes were used to determine the ability of frozen/thawed sperm from subfertile and fertile TB stallions to bind to the ZP. Ovaries were procured from a slaughterhouse 1 h from our laboratory, and the specimens were maintained at 37 °C in an insulated container for transport. Upon arrival at our laboratory, the ovaries were washed with warm PBS and dried with cotton gauze. All visible follicles were sliced using a scalpel blade, and the follicular cavity was flushed using Vigro^®^ Complete Flush Medium (Vetoquinol, Pullman, WA, USA) over a sterile Petri dish. Groups of 50 *cumulus*-oocyte complexes (COCs) were identified using a stereoscope and transferred into a Petri dish containing 150-µL droplets of maturation medium [M199 with Earle’s salts, supplemented with 5 mU/mL FSH (Sioux Chemicals, Sioux Center, IA, USA), 10 IU/mL hCG (Chorulon, Merck Animal Health, Rahway, NJ, USA), 10% FBS, and 25 µg/mL gentamicin] under light mineral oil, at 38.2 °C in a humidified atmosphere of 5% CO_2_ for 22 h. After this period, the COCs were placed into a fresh droplet of maturation medium and incubated for an additional 22 h under the same conditions. Following maturation, COCs were denuded of cumulus by repeated pipetting in M199 medium with Hank’s salts containing 10% FBS and 0.5 mg/mL hyaluronidase. Denuded oocytes were considered matured if a polar body was extruded and present at the periphery of the oolemma. To perform the heterologous zona-binding assay, frozen/thawed semen from three fertile and three subfertile TB stallions was thawed, processed by density gradient centrifugation using 40% Redigrad, and resuspended to 30 × 10^6^ sperm/mL in Lac-MW. A total of 20 mature oocytes were placed in 50-µL droplets of Lac-MW, inseminated with 5 × 10^4^ sperm from each stallion, and incubated for 4 h at 38.2 °C in a humidified atmosphere of 6% CO_2_, 5% O_2_, and 89% N_2_. Following the co-incubation period, to remove any loosely bound sperm, the oocytes were washed by repeated pipetting in M199 with Hank’s salts, transferred to a 50-µL droplet of 2% paraformaldehyde in DPBS for 10 min at room temperature and placed into a 50-µL droplet of 2% BSA + 10 µM Hoechst 33,342 in DPBS for 10 min at room temperature. The oocytes were loaded onto polylysine-coated slides, carefully covered with a coverslip, and examined at 400 × using a BX-60 Olympus fluorescence microscope. For each oocyte, the total number of sperm bound to the ZP was quantified and compared between fertile and subfertile TB stallions.

### Statistical analysis

Statistical analyses were performed using commercial software (SAS Version 9.4; SAS Institute, Inc., Corp., Cary, NC, USA). The Shapiro–Wilk test (PROC UNIVARIATE) was conducted to test data distribution. Within periods (T0h, T2h, T4h, or T6h), Student *t*-tests (PROC TTEST) or the Wilcoxon Ranked Sum test (PROC NPAR1WAY) were used to compare the percent of AE in viable sperm (AE-Viable) the immunolocalization of ARSF in sperm from fertile and subfertile stallions, and the number of sperm bound to the ZP between fertile and subfertile stallions. Group differences were set at P < 0.05.

### Supplementary Information

Supplementary Information 1.

Supplementary Information 2.

Supplementary Information 3.

## Data Availability

The DIA-MS data from this study is accessible at the Mass Spectrometry Interactive Virtual Environment (MASSIVE) repository, with accession number ftp://massive.ucsd.edu/v06/MSV000093730/.
