# Supplementary figures and images for: Proteomic analysis of sperm from fertile stallions and subfertile stallions due to impaired acrosomal exocytosis
Source: Sci Rep. 2024 May 30;14:12446. doi: 10.1038/s41598-024-63410-3 (PMC11139894; doi:10.1038/s41598-024-63410-3)

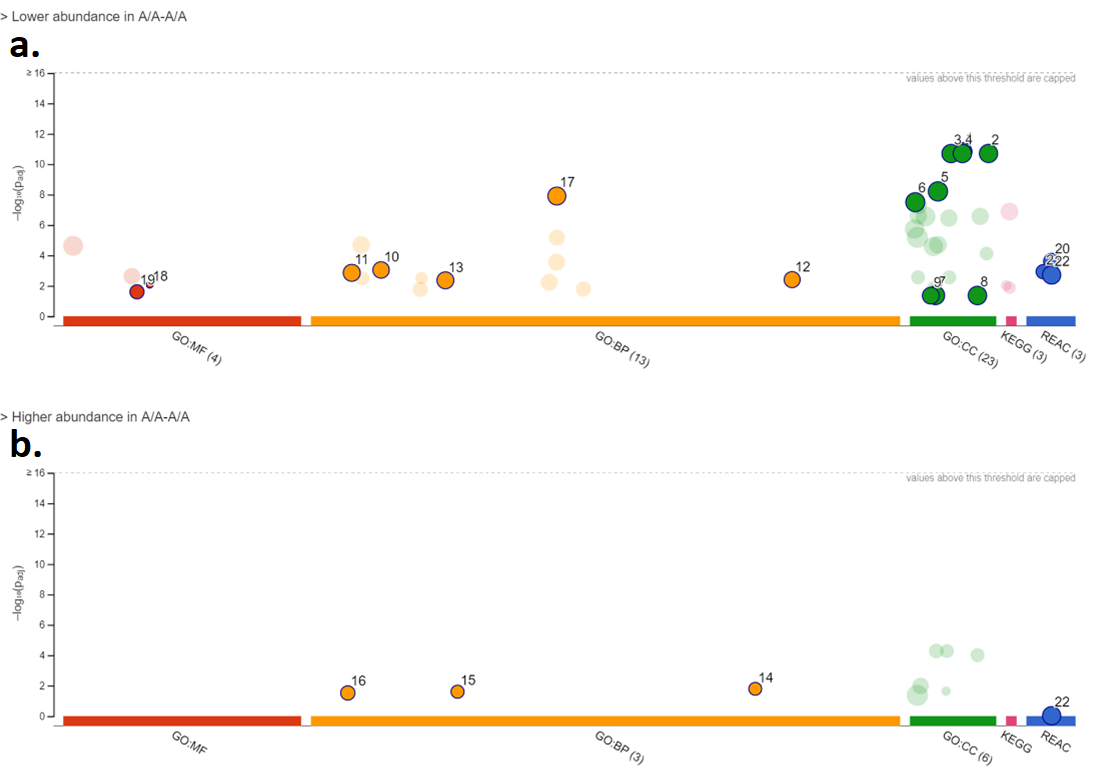

Supplement: Supplementary file 1 — Supplementary Information 1. [file 41598_2024_63410_MOESM1_ESM.tif]

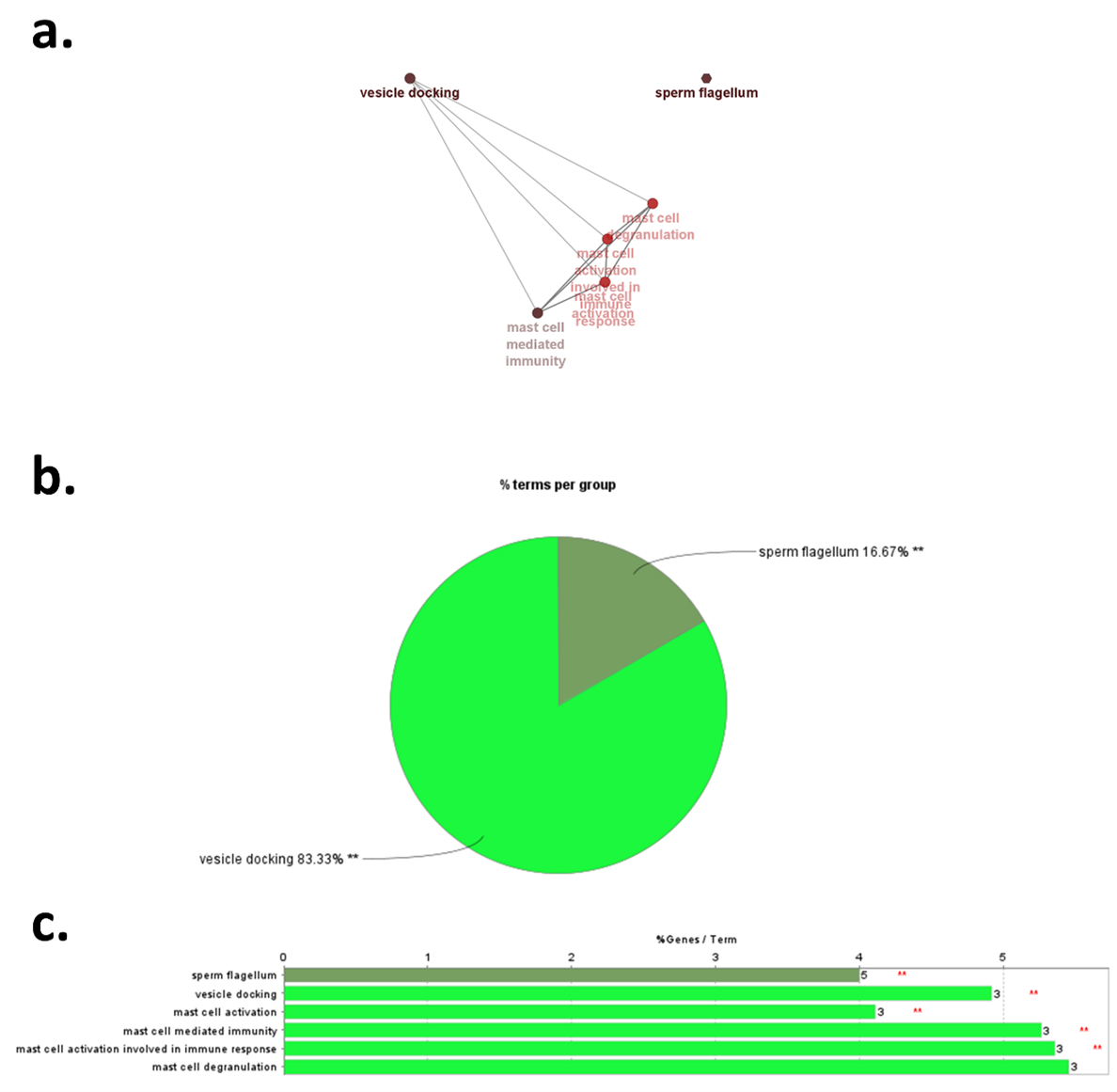

Supplement: Supplementary file 2 — Supplementary Information 2. [file 41598_2024_63410_MOESM2_ESM.tif]
