# Supplementary material for: Proteomic analysis of sperm from fertile stallions and subfertile stallions due to impaired acrosomal exocytosis
Source: Sci Rep. 2024 May 30;14:12446. doi: 10.1038/s41598-024-63410-3 (PMC11139894; doi:10.1038/s41598-024-63410-3)
Supplement: Supplementary file 3 — Supplementary Information 3. [file 41598_2024_63410_MOESM3_ESM.docx]

**Supplementary table 1.** Sperm quality parameters in fresh semen, fertility indices (Mare Book, PC-PR, and SPR), and *FKBP6* genotypes of three fertile (stallions A, B, C) and three subfertile TB stallions (stallions D, E, F) used for this study.

| Stallion | TSN  (x 10^9^) | TMOT  (%) | PMOT  (%) | Normal  (%) | Viable  (%) | COMP_a-t_  (%) | Mare  Book (n) | PC-PR  (%) | SPR  (%) | *FKBP6*  Genotype |
| --- | --- | --- | --- | --- | --- | --- | --- | --- | --- | --- |
| A | 7.6 | 82 | 58 | 66 | 84 | 15 | 31 | 61 | 91 | G/G-A/A |
| B | 8.2 | 85 | 60 | 60 | 82 | 9 | 129 | 63 | 89 | A/G-A/A |
| C | 5.9 | 85 | 50 | 62 | 79 | 6 | 176 | 62 | 90 | G/G-A/C |
| D | 6.4 | 82 | 59 | 52 | 84 | 10 | 51 | 5 | 4 | A/A-A/A |
| E | 6.9 | 87 | 55 | 71 | 85 | 13 | 48 | 29 | 52 | A/A-A/A |
| F | 6.8 | 80 | 60 | 56 | 86 | 10 | 23 | 39 | 61 | A/A-A/A |

TSN = Total sperm numbers in the ejaculate; TMOT = Total motility; PMOT = Progressive motility; Normal = Morphologically normal sperm; COMP_a-t_ = Cells Outside the Main Population; Viable = Sperm with intact plasma membrane; Mare Book = Number of mares bred during the breeding season; PC-PR = Per-cycle pregnancy rate; SPR = Seasonal pregnancy rate.

**Supplementary table 2.** List of gene ontology (GO) terms related to proteins with lower and higher relative abundance in sperm from subfertile TB stallions when compared to sperm from fertile TB stallions. The proteins were queried using *Homo sapiens* orthologs. The ID numbers correspond to the IDs represented in the Manhattan plots in Figure 4. GO: Gene ontology; CC: Cellular component; BP: Biological process; MF: Molecular function; KEGG: Kyoto Encyclopedia of Genes and Genomes; REAC: Reactome.

| Proteins of lower relative abundance in sperm from subfertile TB stallions | | | | |
| --- | --- | --- | --- | --- |
| ID | Source  (GO term) | Term ID | Term Name | *p*-value (adjusted) |
| 1 | CC | GO: 0070062 | Extracellular exosome | 1.480 x 10^-11^ |
| 2 | CC | GO: 1903561 | Extracellular vesicle | 1.975 x 10^-11^ |
| 3 | CC | GO: 0043230 | Extracellular organelle | 2.001 x 10^-11^ |
| 4 | CC | GO: 0065010 | Extracellular membrane-bounded organelle | 2.001 x 10^-11^ |
| 5 | CC | GO: 0031982 | Vesicle | 6.166 x 10^-9^ |
| 6 | CC | GO: 0005615 | Extracellular space | 3.155 x 10^-8^ |
| 7 | CC | GO: 0031410 | Cytoplasmic vesicle | 4.284 x 10^-2^ |
| 8 | CC | GO: 0097708 | Intracellular vesicle | 4.332 x 10^-2^ |
| 9 | CC | GO: 0030141 | Secretory granule | 4.458 x 10^-2^ |
| 10 | BP | GO: 0008610 | Lipid biosynthetic process | 9.091 x 10^-4^ |
| 11 | BP | GO: 0006082 | Organic acid metabolic process | 1.395 x 10^-3^ |
| 12 | BP | GO: 1901615 | Organic hydroxy compound metabolic process | 3.924 x 10^-3^ |
| 13 | BP | GO: 0019752 | Carboxylic acid metabolic process | 4.371 x 10^-3^ |
| 17 | BP | GO: 0044281 | Small molecule metabolic process | 1.247 x 10^-8^ |
| 18 | MF | GO: 0019145 | Aminobutyraldehyde dehydrogenase activity | 8.322 x 10^-3^ |
| 19 | MF | GO: 0016853 | Isomerase activity | 2.493 x 10^-2^ |
| 20 | REAC | REAC:R-HSA-556833 | Metabolism of lipids | 2.530 x 10^-4^ |
| 21 | REAC | REAC:R-HSA-8978868 | Fatty acid metabolism | 1.179 x 10^-3^ |
| 22 | REAC | REAC:R-HSA-1430728 | Metabolism | 1.952 x 10^-3^ |
| Proteins of higher relative abundance in sperm from subfertile TB stallions | | | | |
| 15 | BP | GO: 0140056 | Organelle localization by membrane tethering | 1.615 x 10^-2^ |
| 16 | BP | GO: 0022406 | Membrane docking | 2.505 x 10^-2^ |
| 17 | BP | GO: 0003341 | Cilium movement | 3.018 x 10^-2^ |
